# Emergency care utilization and patients’ outcome before and after COVID-19 national lockdown in Iran: a cross-sectional study

**DOI:** 10.1186/s12873-023-00887-7

**Published:** 2023-09-29

**Authors:** Vahid Ghanbari, Alireza Khatony, Maryam Janatolmakan, Shahab Rezaeian, Leili Rostamnia

**Affiliations:** 1 School of Nursing and Midwifery, Kermanshah University of Medical Sciences, Kermanshah, Iran; 2 Social Development and Health Promotion Research Center, Health Institute, Kermanshah University of Medical Sciences, Kermanshah, Iran; 3 Infectious Diseases Research Center, Kermanshah University of Medical Sciences, Kermanshah, Iran; 4 Clinical Research Development Center, Imam Reza Hospital, Kermanshah University of Medical Sciences, Kermanshah, Iran

**Keywords:** COVID-19, Pandemic, Healthcare, Emergency Department, Emergency Care

## Abstract

**Introduction:**

COVID-19 rapidly spread throughout the world. Stay-at-home and social distance strategies accompanied by fear of contamination with COVID-19 caused significant disruptions in daily life. The study focused on the impact of the COVID-19 pandemic on emergency visit and patients’ outcome in the emergency department (ED).

**Method:**

Administrative and clinical data of 25-hospital EDs in Kermanshah province of Iran from February 20, 2020, to February 18, 2021, were retrospectively analyzed with the comparable periods in the previous year. The incidence rate ratio (IRR) was used to compare the differences between the pandemic and the pre-pandemic period.

**Result:**

The number of ED visits decreased nearly 50% after the declaration of a national lockdown. Moreover, the proportion of patients triaged in ESI 1 and 2 levels increased by 40 and 52%, respectively. The ratio of patients admitted to intensive care units and discharged against medical advice also increased significantly.

**Conclusion:**

Despite the number of ED visits sharply declining, the ratio of patients who came to EDs with higher acuity significantly increased. So, health authorities must sensitize the public about life-threatening signs and symptoms in such conditions.

**Supplementary Information:**

The online version contains supplementary material available at 10.1186/s12873-023-00887-7.

## Introduction

A severe acute respiratory syndrome coronavirus 2 (SARS-CoV-2) emerged from the Wuhan, China in late December 2019 [1]. Then, this viral disease called coronavirus disease 2019 (COVID-19) and spread rapidly throughout the china and internationally (firstly Iran and Italy) [1]. On March 11, 2020, the World Health Organization has declared the COVID-19 disease as a global pandemic [2]. So, governments worldwide responded by implementing various restriction strategies, such as stay-at-home orders, limitations on public gatherings, and social distancing, to control the disease’s spread [3–5]. Moreover, news related to COVID-19 on social media enhanced public concerns [6]. Additionally, health authorities advised people to avoid visiting Emergency Departments (EDs) unless they faced serious health problems [7, 8].

Data from the H1N1 pandemic showed increased ED visits and worsened ED performance indicators like patient stay length, waiting time, and boarding time. [9]. But, these restrictions and warnings led to sharp decreases in ED visits, as people avoided the ED for fear of getting COVID-19 [1, 7]. Research from various countries, including the USA, Israel, Australia, Canada, and Italy, reports significant declines in ED visits for conditions like heart attacks, strokes, obstetric needs, mental health, and pediatrics during the early weeks of the pandemic [1, 3–7, 10–12].

This decline in ED visits has had serious consequences. For instance, Wong et al. reported a significant increase in out-of-hospital cardiac arrests in New York City during the initial COVID-19 surge and a significant delay in hospital arrival for ischemic stroke patients [13]. Similarly, Kugelman et al’s findings suggest that during the COVID-19 period, there was a significant increase in the rate of women visiting emergency departments with premature rupture of membranes and active labor. The author concluded this delay led higher risks for both mother and baby, which could contribute to higher mortality [6]. Gutovitz et al’s finding that mortality of all causes for emergency department visits increased significantly during the COVID-19 pandemic [1]. Then, the increased rates of complications requiring emergency department care and higher associated mortality during the COVID-19 pandemic indicate that delays in seeking emergency care can have serious health consequences. So, the risk of adverse outcomes and mortality is increasing for preventable morbidity and mortality in the future [3, 6, 7]. This issue caused healthcare authorities to worry about the health consequences of delays in requesting emergency care services [6].

Although the initial effects of the COVID-19 pandemic on ED visits for emergency care seeking were understood [4], its long-term consequence on patients’ acuity and the outcome has been less understood. This study focused on the impact of COVID-19 pandemic on the overall ED presentations and patients’ severity of illness and health outcomes. Emergency and disaster planner can use from the results of the present investigation, in order to implement an evidence-based plan for responding to the future pandemic. This retrospective investigation was done using routine data which are gathered by the University of Medical Sciences which is the responsible organization for health in each province of Iran. The paper is divided into five main sections, including introduction, method, result, discussion, and conclusion.

## Method

This cross-sectional retrospective study was conducted by the reported administrative and clinical ED data from 25 hospitals to treatment deputy and Medical Care Monitoring Center (MCMC) of Kermanshah University of Medical Science (KUMS). KUMS is a responsible health organization in Kermanshah Province, located in the west of Iran. Seven of the included hospitals are academic medical centers (trauma, ST-elevation myocardial infarction (STEMI), poisoning, general, pediatric, psychiatric, obstetrics, and gynecology center). The remaining hospitals are governmental or private community hospitals.

The study period was two comparative periods, “a pandemic period” from February 20, 2020, to February 18, 2021, and “a pre-pandemic period” from February 20, 2019, to February 19, 2020. The data set included total ED visits, EMS referral patients, the Emergency Severity Index (ESI) triage priority level, out-of-hospital cardiac arrest, number of CPR, CPR success, death in the 24-first hours, and emergency operations. Regarding the ESI triage, this triage algorithm categorizes patients into five levels, ranging from Level 1 (most severe) to Level 5 (least severe). The levels are determined based on the patient’s presenting symptoms, vital signs, and potential for deterioration and needed resources in the emergency department [14]. The number of patients visiting EDs with chest pain, myocardial infarction (MI), stroke activation code, and gastrointestinal complaints were also gathered. The number of trauma patients and patients referred because of Motor vehicle collision (MVC) were also included in the data set. According to the National and Sub-national Burden of Diseases Atlas, Islamic Republic of Iran 1990–2015, it has been identified that cardiovascular disease, trauma caused by motor vehicles and unintentional injuries, gastrointestinal disease, and cerebrovascular disease are main causes of morbidity and mortality in Iran [15]. In response to these findings and to better address these health challenges, emergency organization managers in Iran have decided to gather daily statistics on these diseases. Furthermore, the final status of patients (admitted in medical wards, intensive wards, and discharge against Medical Advice) from ED was also obtained.

For each of the outcomes, the incidence rate was calculated as the number of the outcomes of interest divided by the total ED visits in each period. To characterize how each of the outcomes was changed during the pandemic period compared with pre-pandemic period, the difference between the percentages of the outcome was calculated. In other words, the difference (%) is compared the raw number of cases between the pre-pandemic and pandemic periods. Poisson regression was also used to calculate the incidence rate ratios (IRR) with 95% confidence intervals to characterize changes in the proportion of case types seen during the COVID-19 period. IRR was calculated by dividing the incidence rate in the early pandemic period by the incidence rate in the baseline period for each outcome. Statistical analysis was performed by Stata 14 (Stata Corp, College Station, TX). The institutional review board of KUMS approved (IR.KUMS.REC.1400.054) and supervised all steps of this research project.

## Result

During the year before the COVID-19 pandemic happen, there were more than two million ED visits in all of the hospitals under the supervision of KUMS. During the first year of the pandemic, the number of ED visits decline to nearly one million. The statistical analysis indicated the number of ED visits sharply decreased (50%) after the national declaration of the COVID-19 pandemic. Although ED visits slightly increased after the first quarter of the pandemic era, the number of ED visits nearly stabilized during the first year of the pandemic (Table 1). Regarding the mode of arrival to ED, the number of patients referred to ED decreased by more than 50% rather than in the pre-pandemic era. Furthermore, the ratio of patients referred to ED by their family or personally declined 12% in comparison to the pre-pandemic period. Surprisingly, the number patients referred by EMS systems declined, but the ratio of referred patients by EMS increased more than 50%. What is unexpected is, the number of patients referred from other hospitals tripled. This is a disappointing outcome that the ratio of patients brought to EDs without vital signs nearly doubled in the COVID-19 period rather than the pre-pandemic period. This difference was greater in the third and fourth quarters of the COVID-19 era.

**Table 1:** Comparison of overall ED visit, mode of arrival to ED between the pre and post-pandemic period

	Characteristics	Comparison	Quarter^1st^	Quarter^2nd^	Quarter^3rd^	Quarter^4th^	Total	Differences		IRR^1^ (95%CI^2^)	P-value
Total ED visit		Pre-Pandemic N (%)	507,895	500,730	497,547	501,750	2,007,922	-49.75		0.50 (0.501, 0.503)	0.001
		Pandemic N (%)	214,215	259,434	289,880	245,521	1,009,050				
Arrival mode to ED	Ambulatory	Pre-Pandemic N (%)	498,572 (98.16)	489,513 (97.75)	485,998 (97.67)	493,133 (98.28)	1,967,396 (97.97)		-11.94	0.88 (0.8–0.97)	0.01
		Pandemic N (%)	206,573 (96.43)	245,929 (94.79)	274,112 (94.56)	235,548 (95.93)	962,162 (95.35)				
	EMS	Pre-Pandemic N (%)	7111 (1.40)	8279 (1.65)	8667 (1.74)	6515 (1.29)	30,572 (1.52)		52.97	1.53 (1.27–1.84)	0.001
		Pandemic N (%)	4746 (2.21)	6957 (2.68)	6615 (2.28)	5290 (2.15)	23,608 (2.34)				
	Referred from other Hospitals	Pre-Pandemic N (%)	2212 (0.43)	2938 (0.58)	2882 (0.57)	2102 (0.41)	10,134 (0.50)		347.80	4.48 (3.39–5.91)	0.001
		Pandemic N (%)	2896 (1.35)	6548 (2.52)	9153 (3.15)	4683 (1.9)	23,280 (2.31)				
	Died	Pre-Pandemic N (%)	135 (0.026)	128 (0.025)	122 (0.024)	137 (0.027)	522 (0.026)		188.49	2.88 (1.92–4.33)	0.001
		Pandemic N (%)	119 (0.055)	160 (0.06)	274 (0.094)	208 (0.084)	761 (0.075)				

(1) IRR: Incidence Rate Ratios (2) CI: Confidence interval

The results of the comparison of the numbers and ratio of ESI triage levels are shown in Table 2. What stands out in Table 2 is the markedly increased ratio of patients referred with lower ESI levels 1, 2, and 3. Moreover, the number of patients triaged in levels 4 and 5 declined significantly. The ratio of patients in Levels 1,3, and 5 did not change significantly during the COVID-19 era rather than in the pre-pandemic era (P > 0.05).

**Table 2:** The incidence and number of acuity levels of patients coming to ED and performing CPR in EDs between the pre and post-pandemic period

	Characteristics	Comparison	Quarter^1st^	Quarter^2nd^	Quarter^3rd^	Quarter^4th^	Total	Differences	IRR (95%)	P-value
ESI triage Level	ESI1	Pre-Pandemic N (%)	2334 (0.46)	1819 (0.36)	2174 (0.44)	1605 (0.31)	7932 (0.39)	40.56	1.41 (0.97–2.04)	0.57
		Pandemic N (%)	1179 (0.55)	1436 (0.55)	1583 (0.55)	1380 (0.56)	5578 (0.55)			
	ESI2	Pre-Pandemic N (%)	45,264 (8.9)	45,078 (9)	40,814 (8.2)	40,485 (8.1)	171,641 (8.54)	52.58	1.53 (1.41–1.65)	0.001
		Pandemic N (%)	25,517 (11.9)	35,691 (13.8)	37,698 (13)	32,842 (13.4)	131,748 (13.05)			
	ESI3	Pre-Pandemic N (%)	71,606 (14.1)	83,287 (16.6)	74,479 (15)	70,265 (14)	299,637 (14.92)	8.48	1.08 (1.02–1.16)	0.43
		Pandemic N (%)	32,094 (15)	43,820 (17)	47,928 (16.5)	40,834 (16.6)	164,676 (16.31)			
	ESI4	Pre-Pandemic N (%)	256,620 (50.5)	245,090 (48.9)	255,940 (51.4)	263,627 (52.5)	1,021,277 (50.86)	-10.94	0.89 (0.86–0.92)	0.049
		Pandemic N (%)	105,525 (49)	115,591 (44.5)	127,917 (44.1)	106,585 (43.4)	455,618 (45.15)			
	ESI5	Pre-Pandemic N (%)	132,073 (26)	116,456 (23.2)	124,040 (25)	125,768 (25.1)	498,337 (24.81)	-1.28	0.99 (0.94–1.04)	0.875
		Pandemic N (%)	49,827 (23.3)	62,085 (24)	74,515 (25.7)	60,866 (24.8)	247,293 (24.5)			
CPR	Total CPR	Pre-Pandemic N (%)	461 (0.09)	444 (0.09)	503 (0.1)	503 (0.1)	1911 (0.09)	100.77	2.01 (1.6–2.51)	0.001
		Pandemic N (%)	407 (0.19)	471 (0.18)	572 (0.2)	472 (0.19)	1922 (0.19)			
	Successful CPR	Pre-Pandemic N (%)	159 (34.5)	138 (31.1)	163 (32.4)	173 (34.4)	633 (33.12)	-14.25	0.86 (0.74–0.99)	0.04
		Pandemic N (%)	115 (28.3)	131 (27.9)	154 (26.9)	137 (19)	537 (28.1)			

The total number of ED CPR remained largely unchanged (Table 2), while the IRR of total CPR was 2.01(1.6–2.51). In other words, the incidence rate of total CPR in the pandemic period is 2 times higher than in the pre-pandemic period. This change remained nearly constant for the whole period of COVID-19. The number of successful CPRs was slightly decreased. Also, the IRR of successful CPR was 0.86 (0.74–0.99); that is, the incidence rate of successful CPR in the pandemic period is 14% lower than in the pre-pandemic period. The most significant difference between successful CPR in pre-pandemic and pandemic era happened in the fourth quarter. This period was concurrent with one of the deadliest peaks of COVID-19 in Iran.

While the proportion of patients admitted to EDs had significantly increased during the pandemic period, the patients’ hospitalization ratio in medical wards had not changed significantly importantly. As shown in Table 3, the proportion of patients admitted to intensive care units, dying during the first day of hospitalization, and discharged against medical advice (DAMA) rose comparably after the declaration of the COVID-19 pandemic (P < 0.05).

**Table 3:** Comparison of destination of patients who visit the EDs during the pre and post-pandemic period

Characteristics	Comparison	Quarter^1st^	Quarter^2nd^	Quarter^3rd^	Quarter^4th^	Total	Differences	IRR (95%)	P-value
Discharge from ED	Pre-Pandemic N (%)	406,061 (79.9)	372,151 (74.3)	397,222 (79.8)	410,455 (81.8)	1,585,889 (78.98)	-12	0.88 (0.80, 0.97)	0.007
	Pandemic N (%)	156,116 (72.8)	177,749 (68.5)	203,096 (70.1)	164,296 (67)	701,257 (69.5)			
Hospitalized in ED	Pre-Pandemic N (%)	101,699 (20)	119,451 (23.9)	100,103 (20.1)	91,158 (18.1)	412,411 (20.54)	30.32	1.3 (1.1–1.54)	0.001
	Pandemic N (%)	52,741 (24.6)	72,124 (27.8)	77,503 (26.7)	68,892 (28.1)	271,260 (26.88)			
Hospitalized in medical wards	Pre-Pandemic N (%)	27,473 (5.41)	34,264 (6.8)	29,418 (5.9)	28,017 (5.6)	119,172 (5.93)	0.78	1.01 (0.87–1.17)	0.92
	Pandemic N (%)	16,325 (7.6)	22,637 (8.7)	22,121 (7.6)	17,833 (7.2)	78,916 (7.82)			
Hospitalized in Intensive Units	Pre-Pandemic N (%)	2336 (0.46)	2953 (0.59)	3039 (0.61)	2980 (0.59)	11,308 (0.56)	43.09	1.43 (1.24–1.65)	0.001
	Pandemic N (%)	2434 (1.13)	2694 (1.03)	2719 (0.93)	2728 (1.1)	10,575 (1.04)			
Die within the first 24 h	Pre-Pandemic N (%)	310 (0.06)	305 (0.06)	340 (0.07)	328 (0.07)	1283 (0.06)	133	2.19 (0.93, 5.14)	0.071
	Pandemic N (%)	295 (0.14)	344 (0.13)	441 (0.15)	333 (0.13)	1413 (0.14)			
Discharge Against Medical Advice (DAMA)	Pre-Pandemic N (%)	7849 (1.5)	7852 (1.6)	7696 (1.5)	7285 (1.5)	30,682 (1.52)	51.86	1.22 (1.06–1.41)	0.01
	Pandemic N (%)	5126 (2.4)	6679 (2.6)	6133 (2.11)	5383 (2.2)	23,321 (2.31)			

The proportion of the patients referring to EDs with chest pain had increased in pandemic period rather than in the pre-pandemic period but the proportion of MI in the pandemic period decreased; however, this change was not significant (P > 0.63). During the pandemic period, the number of stroke code activations rose significantly, especially in the third quarter (P_=_0.001) (Table 4). The number and the proportion of patients who had been referred with gastrointestinal complaints to EDs during the pandemic period noticeably decreased. During the pandemic period, although the number of patients who came to EDs because of trauma (car accidents or other forms of injuries) decreased, the proportion of patients who had come to EDs with trauma and care accidents increased significantly. While the number of the emergency operations decreased dramatically during the pandemic period, the proportion of emergency operations increased noticeably. As shown in Table 4, the greatest decline in the number of emergency operations occurred in the first quarter of the pandemic period.

**Table 4:** Comparison of patients’ chief complaints and emergency operations between the pre and post-pandemic period

Characteristics	Comparison	Quarter^1st^	Quarter^2nd^	Quarter^3rd^	Quarter^4th^	Total	Differences	IRR (95%)	P-value
Chest pain	Pre-Pandemic N (%)	23,615 (4.65)	19,628 (3.9)	22,583 (4.5)	23,868 (4.76)	89,694 (4.46)	41.9	1.42 (1.27–1.59)	0.001
	Pandemic N (%)	15,116 (7.1)	15,808 (6.1)	14,560 (5)	16,518 (6.7)	62,002 (6.14)			
MI Code	Pre-Pandemic N (%)	86 (0.02)	46 (0.01)	41 (0.01)	47 (0.01)	220 (0.01)	-15.02	0.85 (0.44–1.65)	0.63
	Pandemic N (%)	22 (0.01)	35 (0.01)	34 (0.01)	50 (0.02)	141 (0.014)			
Stroke code	Pre-Pandemic N (%)	114 (0.02)	81 (0.02)	79 (0.02)	120 (0.04)	394 (0.02)	180.9	2.81 (1.71–4.63)	0.001
	Pandemic N (%)	89 (0.03)	98 (0.08)	239 (0.04)	113 (0.04)	539 (0.05)			
GI problem	Pre-Pandemic N (%)	20,537 (4)	18,202 (3.6)	16,042 (3.2)	13,576 (2.7)	68,357 (3.4)	-30.8	0.69 (0.59–0.81)	0.001
	Pandemic N (%)	4949 (2.3)	6330 (2.4)	6087 (2.1)	5582 (2.3)	22,948 (2.27)			
Trauma	Pre-Pandemic N (%)	18,801 (3.7)	18,484 (3.7)	21,726 (4.4)	16,885 (3.4)	75,896 (3.8)	63.85	1.64 (1.45–1.85)	0.001
	Pandemic N (%)	13,450 (6.3)	18,901 (7.3)	16,897 (5.8)	12,978 (5.3)	62,226 (6.2)			
Traffic trauma	Pre-Pandemic N (%)	5252 (1)	5357 (1.1)	6368 (1.3)	4377 (0.9)	21,354 (1.1)	48.48	1.48 (1.18–1.87)	0.001
	Pandemic N (%)	3248 (1.5)	5023 (2)	4723 (1.6)	3018 (1.2)	16,012 (1.6)			
Emergency Operation	Pre-Pandemic N (%)	12,302 (2.4)	10,787 (2.2)	11,754 (2.4)	9078 (1.8)	43,921 (2.19)	69.06	1.69 (1.44–1.98)	0.001
	Pandemic N (%)	8189 (3.8)	10,087 (3.9)	9544 (3.3)	8512 (3.5)	36,332 (3.6)			

## Discussion

Consistent with previous research in other countries [1, 4, 16, 17], our result confirmed a critical decline in ED visits during the first years of the COVID-19 pandemic compared to the identical period in 2019. At the beginning of COVID-19, the MOH introduced hospitals as one of the main centers of contamination with the virus. Therefore, people were afraid of exposed to COVID-19 in the ED. Another reason for this decline can be the tendency of people to treat minor diseases and symptoms or seek alternative treatments at home.

Considering the mode of arrival to ED, our finding indicated that although referring by the EMS and from other hospitals significantly increased, ambulatory patients significantly decreased. Conversely, Stella et al. (2020) showed that the number of EMS missions before and after the pandemic was similar [8]. Westgard et al. showed that during 28 days after the emergency declaration, all types of ED visits (ambulatory and referring by the EMS or police, and fire department) were significantly decreased [3]. However, consistent with the present results, Saberian et al. have demonstrated that after the declaration of the COVID-19 outbreak, the number of EMS missions’ rose by 21% [18]. Further analysis showed that intra-hospital patient transfer was remarkably increased. Lucero et al. (2020) indicated the number of patients who transferred from ED significantly increased in the COVID-19 era [19]. Another important finding is that the number of patients who come to ED without vital signs increased nearly two times. This finding is consistent with Wong et al. who reported out of hospital cardiac arrest was dramatically increased [13]. These results support the idea of postponing seeking emergency care by the community member and they were avoiding ED visits even when they had serious health-threatening situations. This study also found that patients referring to EDs during the pandemic period had a higher acuity level in comparison with the corresponding time. This finding matches those observed in earlier studies [4, 13, 17]. Another important finding indicates the higher-acuity of patients who visit the ED markedly increased the rate of patients with cardiac arrest in ED and significantly decreased the rates of return of spontaneous circulation (ROSC). This finding is in accord with recent studies indicating that the COVID-19 pandemic has significantly increased the rate of cardiac arrest [20, 21].

The result of this research shows that although the proportion of patients admitted to the ED departments and intensive care units has increased markedly during the COVID-19 pandemic, the proportion of hospitalization in medical wards has not changed significantly. Giamello et al. (2020) reported a significant increase in hospitalizations in Cuneo of Italy [22]. Jeffery et al. also indicated that hospital admission from the ED was stable until COVID-19 cases increased [17]. Baugh et al. also reported that a higher percentage of patients were admitted to the Intensive Care Unit (ICU) during COVID-19 Pandemic [4].

Another important finding is that dying within the first 24 h of hospitalization notably increased during the pandemic era. This finding is contrary to Morishita et al. who have suggested that the in-hospital mortality within the 24 first hours did not change during the COVID-19 pandemic [23]. This result may be due to patients who came to ED with a higher acuity level. Furthermore, this study found that the proportion of patients who left the ED against medical advice had noticeably increased during the COVID-19 pandemic. Aydin et al. reported during COVID-19 pandemic DAMA was increased 24.5%. Demir et al. (2021) reported the most common reasons for DAMA in COVID-19 were the fear of being infected by COVID-19, and the thought of being neglected [24]. Further research is required to evaluate the impact of DAMA on patients’ outcomes.

Another important finding is that the proportion of patients who come to ED with chest pain increased during the pandemic period. However, the number and the proportion of patients who had a MI had decreased. These results are in keeping with previous observational studies, which reported a significant decline in the admission of patients with acute coronary syndrome [5, 25, 26]. Several factors could explain this observation. Firstly, signs and symptoms like chest discomfort and dyspnea can be easily misinterpreted as being related to COVID-19. Secondly, the frequent message about staying at home. Finally, the fear of COVID infection in an ED may lead patients with an ACS to defer seeking medical care. Surprisingly, contrary to previously published studies [5, 11, 26], the study result shows that the activation code of stroke notably increased during the pandemic period. A possible explanation for this finding might be that occurrence of the pandemic led to the limitation of access to health care. Therefore, such patients could not follow their treatment protocol or visit their physician regularly. As a result, their health status was not under control. Furthermore, this finding confirms the previously mentioned result of the study that indicates patients come to ED with a higher acuity situation. This is an important issue for future research.

The results of this investigation show a clear-cut decline in the number and proportion of patients who come to EDs with GI complaints. This finding is in line with those of previous studies [3, 4, 22, 27]. The study result also demonstrated a steep decline in the number of patients referred to EDs with trauma (MVCs 48% and other types of trauma 63%) during the pandemic period rather than the comparison period. This finding is consistent with Boserup et al., which demonstrated the declaration of the COVID-19 pandemic led to a significant decrease in MVCs in the USA [16]. This finding could be explained by the implementation of national lockdown during the four peaks of COVID-19 in the first year of the pandemic in Iran and the rise in the distance working and extreme decrease in recreational or working trips. Surprisingly the ratio of trauma patients rather than the total number of patients referred to EDs increased during the pandemic period.

In addition, there was a significant increase in the proportion of emergency operations that were performed in the pandemic era. Baugh et al. reported that despite the decrease of some bedside procedures (such as laceration repair), the number of emergency laparotomies increased significantly [4]. The reasons for this result are changing the health system for admission of elective patients and delayed presentations of surgical conditions such as appendicitis or cholecystitis.

### Limitations

This retrospective study utilized aggregated data from an administrative database that collects administrative and clinical information in KUMS and is therefore subject to potential data inconsistencies. This study only includes EDs data from hospitals in the catchment area, and no special 16-hour health centers were established by the health system during the COVID-19 pandemic.

## Conclusions

The number of ED visits remarkably decrease after the declaration COVID pandemic and this decline remain during the first year of this health emergency. It is also found that the proportion of patients who come to EDs with higher acuity situations significantly increased. In light of these findings, it is important for health authorities must sensitize people to serious symptoms, illnesses, and injuries that cannot be managed in other settings. Furthermore, healthcare organizations should have a defined plan to confront the public’s fear of disease contagion at EDs in future pandemics. Defining dirty and clean areas in EDs and informing patients may also convince people about coming to an ED during a pandemic. In addition, further research will also be required to evaluate the impact of these changes in seeking emergency care on the long-term morbidity and mortality in the communities.

### Electronic supplementary material

Below is the link to the electronic supplementary material.

Supplementary Material 1

## Data Availability

The datasets generated and/or analyzed during the current study are not publicly available due to participant confidentiality but are available from the corresponding author on reasonable request.

## Abbreviations

ED: Emergency Department
IRR: Incidence Rate Ratio
ESI: Emergency Severity Index
MCMC: Medical Care Monitoring Center
KUMS: Kermanshah University of Medical Science
STEMI: ST-Elevation Myocardial Infarction
EMS: Emergency Medical System
MVC: Motor vehicle collision
CPR: Cardio-Pulmonary Resuscitation
